# Aggressive renal angiomyolipoma with vena cava extension: A case report and literature review

**DOI:** 10.3892/ol.2014.2428

**Published:** 2014-08-08

**Authors:** GANG SHEN, QIQI MAO, HANJIN YANG, CHAOJUN WANG

**Affiliations:** 1 Department of Urology, First People’s Hospital of Wujiang District, Medical School of Nantong University, Suzhou, Jiangsu 215200, P.R. China; 2 Department of Urology, The First Affiliated Hospital, School of Medicine, Zhejiang University, Hangzhou, Zhejiang 310003, P.R. China; 3 Department of Pathology, The First Affiliated Hospital, School of Medicine, Zhejiang University, Hangzhou, Zhejiang 310003, P.R. China

**Keywords:** angiomyolipoma, thrombosis, inferior vena cava

## Abstract

Renal angiomyolipoma (AML) is the most common type of benign mesenchymal tumor of the kidney. AMLs typically present as benign lesions without local invasion. However, the tumor may exhibit aggressive behavior. Intravascular extension into the inferior vena cava (IVC) and hemorrhagic aneurysm formation associated with AML has rarely been reported in the past. In the current study, the novel case of a 77-year-old female is described who presented with a tumor thrombus extending to the IVC. The patient subsequently underwent a radical nephrectomy and an IVC tumor thrombectomy. In addition, the available literature regarding this unusual complication of a common renal neoplasm has been reviewed. It is essential for radiologists and clinicians to be aware that AMLs may exhibit these types of aggressive behaviors.

## Introduction

Renal angiomyolipomas (AMLs) are usually benign lesions, however, liposarcomatous degeneration has previously been described (1). AMLs may occur sporadically or may be associated with tuberous sclerosis (TS) when they are bilateral and multifocal. The cells of AMLs are derived from perivascular epithelioid cells, which are composed of variable quantities of blood vessels, smooth muscle and mature adipose tissue (2). The involvement of the renal vein, inferior vena cava (IVC) and regional lymph nodes has rarely been reported. In the current study, a case of a large AML presenting with an aggressive tumor thrombus extension into the intrahepatic IVC is described; a review of the literature was also performed. The patient’s families provided informed consent.

## Case report

**Figure 1:**
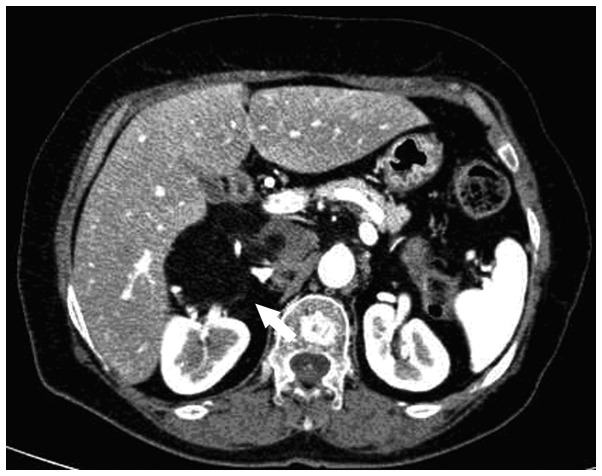
Contrast multi-slice computed tomography scan revealed a right fat-containing renal mass with renal vein and vena caval extension.

A 77-year-old female patient was incidentally diagnosed with a tumor of the right kidney during a health examination by ultrasound at the Department of Urology, First Affiliated Hospital (Hangzhou, China) on September 10, 2013. There was no history of right flank pain, dysuria, gross hematuria, abdominal distention, pyrexia or lower extremity edema. The patient had been diagnosed with hypertension five years previously and was receiving regular medication. The family history of TS was negative and a physical examination and laboratory tests revealed no abnormalities. Ultrasonography revealed a hyperechoic mass at the lower pole of the right kidney, which extended into the IVC. Furthermore, a contrast-enhanced abdominal computed tomography (CT) scan revealed an extensive mixed hypodense mass, which was predominantly composed of fat (attenuation, −79 HU; Fig. 1). CT angiography identified a thrombus-like lesion in the renal vein extending into the IVC up to the level of the hepatic vein. Bone scans and chest X-rays were negative for metastases.

**Figure 2:**
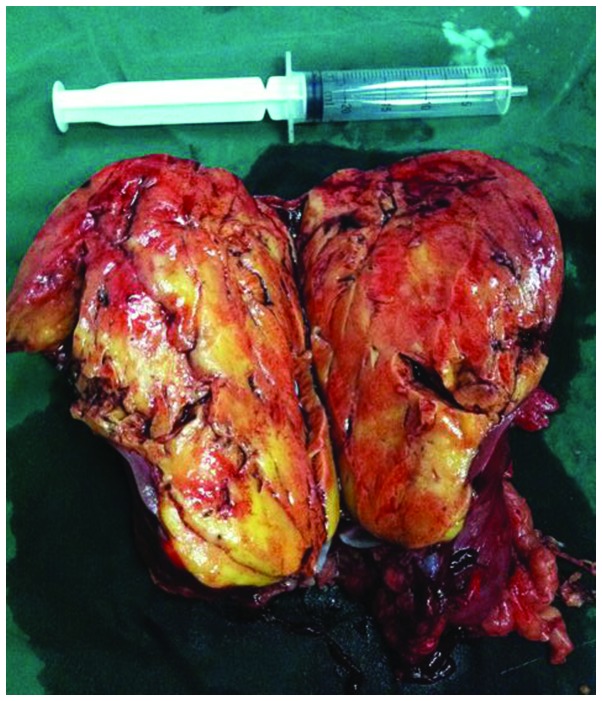
Gross specimen of the right kidney with fatty tumor and yellow thrombus.

**Figure 3:**
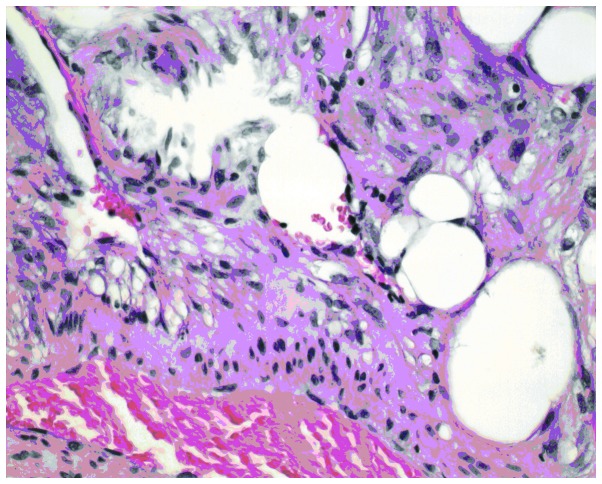
Microscopic appearance of the tumor revealing the typical mixture of mature adipose cells, thick-walled vessels and smooth muscle cells, which stained positively for human melanoma black-45 (staining, hematoxylin and eosin; magnification, ×400).

The patient was diagnosed with a large right renal AML with an IVC tumor thrombus, and subsequently underwent a right radical nephrectomy and IVC thrombectomy via a right subcostal incision. No infiltration of the thrombus into the venous wall or enlarged lymph nodes was identified during surgery. The gross specimen section exhibited a yellow mass, which was invading the perirenal tissues (Fig. 2). The tumor thrombus presented as a long strip with a fat-like appearance. Histopathological examination of the tumor revealed the appearance of a classical AML, exhibiting mature adipose tissue, smooth muscle cells and thick-walled blood vessels. The tumor exhibited infiltrative growth with regional cellular atypia and the tumor thrombus was composed of adipose tissue (Fig. 3). Pathological analysis determined the diagnosis of AML with a caval thrombus. Human melanoma black-45, smooth muscle actin and Melan-A were identified to be positive during immunohistochemical analysis. The histological evidence did not indicated that the tumor was malignant. Postoperatively, the patient was admitted to the intensive care unit of the First Affiliated Hospital (Hangzhou, China)and subsequently transferred to an ordinary ward following two days of observation. The patient was discharged following an uneventful postoperative period.

## Discussion

AMLs, also termed hamartomas, are composed of large quantities of mature adipose tissue, smooth muscle tissue and thick-walled blood vessels. AML is a type of perivascular epithelioid cell tumor and a total of 20% of AMLs are associated with TS. Furthermore, AMLs that are associated with TS are frequently bilateral and multifocal. The first case of AML presenting with intravenous thrombus was reported by Kutcher *et al* in 1982 (3). To the best of our knowledge, 34 cases of AML presenting with intravascular tumor growth have been reported, which includes six cases in males (4–9) and the remaining cases were in female patients. The majority of cases occurred as sporadic isolated cases, although two cases were associated with TS. The patients were aged between 42 and 50 years; therefore, a unique aspect of the present case was the age of the patient who was 77 years old. Furthermore, the tumor was particularly large and did not exhibit any clinical symptoms.

Conventional AMLs are typically asymptomatic in the early stage with an imperceptible onset and slow growth. The detection of AML may be incidental during the course of a routine inspection or health check-up. When the tumor grows larger, the presence of an AML may be indicated by lower back discomfort, abdominal pain or other symptoms, and the likelihood of a spontaneous rupture and life-threatening hemorrhage is significantly increased. Epithelial AML (EAML), a subtype of AML, is considered to be a unique type of renal tumor with malignant potential (10). Luo *et al* (11) reported a small number of cases of kidney EAML with IVC involvement.

Early diagnosis of AML predominantly relies on imaging techniques. Ultrasound is an effective screening method and CT provides the greatest diagnostic value. Typically, AMLs are rich in fat and represented on CT scans by a low signal, rendering them easy to diagnose (12). Approximately 5% of AMLs lack fat and, therefore, cannot be differentiated from renal cell carcinomas (RCCs). Therefore, for AMLs which lack fat and thus, are difficult to diagnose by CT, previous studies have proposed that MRI may improve the accuracy of diagnosis with the use of fat saturation techniques (13,14). Annual imaging examinations are proposed for patients with sporadic tumors measuring <4 cm. However, for large tumors (≥4 cm), the majority of previous studies recommend surgical treatment, even for benign tumors. Transarterial selective embolization and nephron sparing surgery are suitable treatment strategies for these large tumors. However, AML with IVC involvement may also require surgical treatment even though it is asymptomatic (6,9). As tumor thrombi are fragile, there is a risk of fatal heart embolism prior to and during surgery. Therefore, radical nephrectomies and IVC thrombectomies are advised. Certain studies have performed laparoscopic surgery and achieved favorable results.

AML is considered to be a benign neoplasm and is associated with a favorable prognosis. However, a rare case of renal AML presenting with liposarcomatous transformation has previously been reported (1). Yiu *et al* (15) summarized sixteen studies and identified no association between aggressive behavior and tumor size. Eble (2) identified 20 AML cases that presented with an IVC thrombus or regional lymph node involvement, and demonstrated that the resected tumors, thrombus and lymph nodes were all benign and did not exhibit recurrence or progression during follow-up. Following a review of 35 cases in the literature, Tan *et al* (9) hypothesized that when an AML exhibits aggressive behavior, malignant transformation must have occurred.

In conclusion, it is essential that urologists consider the potential presentation of AML involving the renal vein and/or IVC. In addition, it is proposed that the risk and benefits of surgical treatment are discussed prior to surgery. However, increased attention on establishing a variety of methods for treating this type of lesion is required.
